# Tolerability and efficacy of *Mycobacterium avium* complex pulmonary disease treatment in elderly patients

**DOI:** 10.1186/s12890-025-03504-4

**Published:** 2025-02-07

**Authors:** Kyota Shinfuku, Hiromichi Hara, Keitaro Okuda, Hanae Miyagawa, Naoki Takasaka, Takeo Ishikawa, Jun Araya

**Affiliations:** 1 Division of Respiratory Diseases, Department of Internal Medicine, The Jikei University Daisan Hospital, 4-11-1 Izumihoncho Komae, Tokyo, 201-8601 Japan; 2 Division of Respiratory Diseases, Department of Internal Medicine, The Jikei University School of Medicine, Tokyo, Japan

**Keywords:** Elderly patients, Tolerability, Efficacy, *Mycobacterium avium* complex pulmonary disease, Nontuberculous mycobacteria

## Abstract

**Background:**

*Mycobacterium avium* complex pulmonary disease (MAC-PD) is considered to be increasing worldwide. In Japan, the number of elderly MAC-PD patients requiring treatment is also expected to increase due to the aging society. However, reduced organ function in elderly patients makes it often difficult to continue or complete multidrug treatment due to adverse drug reactions (ADRs). Therefore, this study aimed to identify clinical factors associated with treatment tolerability, efficacy, and ADRs in elderly MAC-PD patients.

**Methods:**

We retrospectively reviewed the medical records of 102 patients with MAC-PD aged ≥ 75 years between January 2014 and March 2023. Forty-six patients were treated with multidrug regimens (treatment group), and 56 were observed without treatment (observation group). The treatment group was divided into the treatment continuation group (*n* = 28) who were treated without interruption for ≥ 12 months, and the treatment interruption group (*n* = 18). A comparative study was conducted in each group to examine tolerability, efficacy, and ADRs.

**Results:**

A two-drug regimen of ethambutol (EB) and macrolides without rifampicin (RFP) was associated with treatment continuation (*p* = 0.026). The treatment continuation group was superior to the observation group regarding symptoms change, sputum conversion rate, and chest computed tomography scores. The most common ADRs were gastrointestinal disorders, which may be related to RFP. Treatment efficacy of the two-drug regimen was non-inferior, and no cases of macrolide resistance were observed.

**Conclusions:**

The two-drug regimen of EB and macrolide without RFP may be a tolerable and effective treatment for elderly MAC-PD patients.

## Background

Nontuberculous mycobacteria (NTM) are mycobacteria other than *Mycobacterium tuberculosis* complex and *Mycobacterium leprae*, and comprise over 200 species. NTM are ubiquitous bacteria found in soil, dust, and water, and sometimes causes pulmonary disease (PD) [1]. Members of the *Mycobacterium avium* complex (MAC) is the most commonly isolated NTM respiratory pathogen. Structural changes in lung and immunological susceptibility are associated with MAC-PD development. The incidence and prevalence of MAC-PD have been increasing in Japan [2]. Despite standard multidrug therapy, treatment success rate remains approximately 60%[3] and the recurrence rate after treatment is approximately 40% [4].

Japan’s population is aging rapidly compared to other countries. According to studies by the Ministry of Health, Labor and Welfare, the aging trend is estimated to progress consistently until 2070, with those aged ≥ 65 years accounting for 38.7% of Japan’s population [5]. As the population ages, the number of elderly MAC-PD patients requiring treatment is also expected to increase [2, 6]. 

Current American Thoracic Society (ATS) and British Thoracic Society guidelines recommend that MAC-PD should be treated with a multi-drug regimen, including macrolides, and continued for at least 12 months after sputum conversion [1, 7]. Long-term continuous antibiotic therapy is critical to achieving treatment goal. And, adverse drug reactions (ADRs) associated with multi-drug therapy are the most important issues affecting treatment continuation. Discontinuation rates of MAC-PD treatment due to ADRs varied for each drug, ranging from 17 to 75%, posing a major challenge in the long-term continuation of multi-drug therapy [8]. Elderly patients are more likely to discontinue treatment due to ADRs due to decreased organ function, complications, and drug interactions with other drugs to treat comorbidities [9, 10]. Kim et al. reported that MAC-PD treatment in patients aged ≥ 80 years is often interrupted by ADRs and proposed a reduction in the intensity of treatment in terms of dosage and number of drugs [10]. Miwa et al. reported that a two-drug regimen with ethambutol (EB) and clarithromycin (CAM) was noninferior in efficacy and had a lower rate of ADRs than a three-drug regimen including rifampicin (RFP) [11]; however, this study was not designed for elderly MAC-PD patients. MAC-PD treatment in elderly patients is considered an important issue; however, reports are very scarce. Effective treatment that can be continued even by the elderly MAC-PD patients is important to investigate. This study aimed to determine the clinical factors associated with tolerability, treatment efficacy, and ADRs in elderly MAC-PD patients and investigate optimal treatment for them.

## Methods

### Study participants

This retrospective cohort study included patients diagnosed with MAC-PD based on the official ATS/ERS/ESCMID/IDSA guideline [1] at Jikei University Hospital and Jikei Daisan Hospital between January 1, 2014 (01/01/2014) and March 31, 2023 (03/31/2023). We included patients whose clinical course could be followed up for at least 12 months at the age ≥ 75 years. Based on the definition of elderly people by the Japan Geriatrics Society, this study defined elderly people as those aged ≥ 75 years [12]. Macrolide-resistant MAC-PD was excluded because drug susceptibility to macrolide significantly affects the clinical course. Co-infections with other mycobacteria and combined lung cancer cases were excluded because they affect the clinical course and imaging evaluation. Additionally, patients who discontinued treatment for comorbidities were also excluded.

The treatment group was defined as patients who started a multi-drug regimen at the age ≥ 75 years. Treatment groups were divided into three-drug regimen of RFP, EB, and macrolide (CAM or azithromycin (AZM)) and two-drug regimen of EB and macrolide.

Treatment with aminoglycoside antibiotics in addition to three-drug regimen was excluded due to evaluation complexity. When patients were treated multiple times, the analysis was performed on the treatment initiated at an older age.

The treatment continuation group included cases in which treatment was started and continued ≥ 12 months. Treatment continuation without interruption was important in this study, and cases that continued treatment after adjustments were included in the treatment continuation group; the treatment interruption group included cases in which treatment was started but discontinued within 12 months owing to ADRs; and the observation group included cases in which treatment was not started during the observational period.

### Clinical diagnosis

Respiratory tract specimens were cultured in Ogawa media or Mycobacterium Growth Indicator Tubes, and the cultured mycobacteria were identified by mass spectrometry or DNA-DNA hybridization (DDH) (SRL, Inc., Tokyo, Japan). Since mass spectrometry and DDH cannot distinguish between *Mycobacterium intracellulare* (*M.intracellulare*) subsp. *intracellulare* and *M.intracellulare* subsp. *chimaera*, ther were grouped as *M.intracellulare*. Minimal inhibitory concentrations of clarithromycin was assessed by Broth MIC NTM (Kyokuto Pharmaceutical Industrial Co., Ltd., Tokyo, Japan), and minimal inhibitory concentrations > 32 µg/ml was considered resistant [13]. High-resolution lung computed tomography (CT) was performed at diagnosis and follow-up, and MAC-PD diagnosis was made according to ATS/ERS/ESCMID/IDSA diagnostic criteria [1]. 

### Data collection and outcomes

We retrospectively reviewed the medical records of the enrolled patients. Patient background, age, sex, body mass index (BMI), comorbidities, underlying pulmonary disease, bacterial species, disease type, treatment, and laboratory blood data were examined. Symptoms change was evaluated based on the medical records for cough, sputum, blood sputum, dyspnea, fever, and general malaise and was classified as improvement, stable, or worsening. Sputum conversion was defined as three consecutive negative sputum cultures. Pulmonary imaging was evaluated based on the chest CT scores reported by Kim et al. [14] Five types of lung lesions, bronchiectasis (severity, 3 points; extent, 3 points; mucus plugging, 3 points; total 9 points), cellular bronchiolitis (severity, 3 points; extent, 3 points; total 6 points), cavities (diameter, 3 points; wall thickness, 3 points; extent, 3points; total 9 points), nodule (3 points) and consolidation (3 points), were given CT scores for a total of 30 points. CT scores were evaluated by three respiratory specialists (KS, KO, and HM), each with at least 10 years of experience. CT scores changes were classified as an improvement (score decrease) and as worsening (score increase).

The primary endpoint was to identify clinical factors associated with treatment tolerability in elderly MAC-PD patients. Therefore, clinical factors were compared between the treatment continuation and interruption groups. Secondary endpoints were treatment efficacy and ADRs. Treatment efficacy was compared between the treatment continuation and observation groups in terms of symptoms change, sputum conversion rate, and chest CT scores between diagnosis and final outpatient visit. Comparison was also made based on drug regimen within the treatment continuation group. ADRs that were causally associated with interruptions were examined in the treatment group.

### Statistical analysis

Fisher’s exact and chi-square tests were used for categorical variables and Mann–Whitney U test and t-test for continuous variables. Statistical analysis was performed using GraphPad Prism version 8.4.3, for Macintosh (GraphPad Software La Jolla, CA, USA), and P values < 0.05 were considered statistically significant.

## Results

### Patient characteristics

Between January 2014 and March 2023, 102 patients were included in this study. Forty-six patients were in the treatment group and 56 in the observation group without treatment induction. Patient characteristics are shown in Table 1. The mean age at diagnosis was 78 years in the treatment group and 80 years in the observation group. The observation group had a higher proportion of males (*p* = 0.009), and more cases of chronic obstructive pulmonary disease (*p* = 0.038), and chronic heart failure (*p* = 0.033). Cavitary cases, including fibrocavitary (FC) and FC + nodular bronchiectasis (NB) types, were treated more frequently (*p* < 0.001). CT scores were higher in the treatment group (*p* < 0.001). In the observation group, treatment was not initiated mainly because sputum smear tests were negative or there were no symptoms.

**Table 1:** Patient characteristics

Parameters	Total (*n* = 102)	Treatment group (*n* = 46)	Observation group (*n* = 56)	*p*-value
Age (at diagnosis, years)^a^	79.3 ± 5.4	78.5 ± 4.2	80.1 ± 6.2	0.145
Gender (%Male)^b^	32 (31%)	8 (17%)	24 (42%)	0.009
BMI (kg/m^2^)^a^	19.4 ± 3.1	19.2 ± 2.9	19.5 ± 3.2	0.768
Total follow up time (months)^c^	48 (33–65)	51 (37–79)	42 (28–60)	0.066
Chronic pulmonary diseases^b^				
Interstitial lung diseases	5 (4%)	3 (6%)	2 (3%)	0.655
Chronic obstructive pulmonary diseases	9 (8%)	1 (2%)	8 (14%)	0.038
Old pulmonary tuberculosis	13 (12%)	5 (10%)	8 (14%)	0.767
Bronchial asthma	9 (8%)	2 (4%)	7 (12%)	0.179
Comorbidities^b^				
Chronic renal failure	13 (12%)	5 (10%)	8 (13%)	0.767
Chronic heart disease	13 (12%)	2 (4%)	11 (19%)	0.033
Malignant tumor	27 (26%)	13 (23%)	14 (25%)	> 0.999
Bacterial species (*M.avium* / *M.intracellulare*)^b, †^	86 (84%) / 20 (19%)	37 (80%) / 11 (23%)	49 (87%) / 9 (16%)	0.332
Disease type (NB/ NB + FC/ FC)^b^	77 (75%) / 22 (21%) / 3 (2%)	26 (56%) / 18 (39%) / 2 (4%)	51 (91%) / 4 (7%) / 1 (1%)	< 0.001
Evaluation of chest CT				
CT scores (at diagnosis)^c^	10 (6–15)	14 (9–17)	9 (5–12)	< 0.001
The reason for not initiating treatment				
Smear negative	・・・	・・・	28 (50%)	
Absence of symptoms	・・・	・・・	24 (42%)	
Patient’s request	・・・	・・・	3 (5%)	
Narrow lesion	・・・	・・・	1 (1%)	

Data are presented as ^a^ mean ± SD, ^b^ n (%) or ^c^ median (range quartile)

†: Both *Mycobacterium avium* and *Mycobacterium intracellulare* were detected in four cases

BMI, body mass index; *M.avium*,* Mycobacterium avium*; *M.intracellulare*,* Mycobacterium intracellulare*; NB, nodular bronchiectasis; FC, fibrocavitary

## Clinical factors related to tolerability

Clinical factors related to tolerability were examined in the treatment continuation and interruption group (Table 2). Of the 46 patients in the treatment group, 32 were administered a three-drug regimen (RFP, EB, and macrolide), and 14 were administered a two-drug regimen (EB and macrolide). Of the 14 patients who received two-drug regimen, 7 were judged by each respiratory specialist to have poor tolerance for three-drug regimen. 4 patients were started by two-drug regimen, and ADRs appeared, and RFP was judged difficult to start. 2 patients changed from three-drug regimen to two-drug regimen due to ADRs of RFP. One patient was treated with two-drug regimen to avoid drug interactions that could affect treatment of comorbidities. Of the 46 patients in the treatment group, 28 (60%) were able to continue treatment for > 12 months. No significant differences were observed in patient background, comorbidities, bacterial species, disease type, laboratory data, or CT scores. Although the dosages of each drug were not significantly different in each group, the three-drug regimen with RFP was associated with treatment discontinuation (*p* = 0.026). Continuation rates were higher for the two-drug regimen. In the treatment group, only two patients adjusted from CAM to AZM within the same macrolide antimicrobial drug due to anorexia and taste disorder, and continued without interruption.

**Table 2:** Clinical factors related to tolerability

Parameters	Treatment continuation group (*n* = 28)	Treatment interruption group (*n* = 18)	*p*-value
Age (at the start of treatment, years)^a^	80.3 ± 4.4	80.2 ± 4.0	0.973
Gender (%Male)^b^	5 (17%)	3 (16%)	> 0.999
BMI (kg/m^2^)^a^	19.4 ± 2.5	19.3 ± 2.7	0.978
Time from diagnosis to treatment (days)^c^	196 (50-1197)	148 (41-1287)	0.796
Comorbidities^b^			
Chronic renal failure	3 (10%)	2 (11%)	> 0.999
Chronic heart disease	0 (0%)	2 (11%)	0.147
Malignant tumor	7 (25%)	6 (33%)	0.738
Bacterial species (*M.avium* / *M.intracellulare*)^b, †^	21 (75%) / 8 (28%)	16 (88%) / 3 (16%)	0.487
Disease type (NB/NB + FC/FC)^b^	16 (57%) / 10 (35%) /2 (7%)	10 (55%) / 8 (44%) /0 (0%)	0.915
Treatment			
two-drug regimen without RFP^b^	12 (42%)	2 (11%)	0.026
EB (mg/kg)^a^	12.1 ± 2.1	12.6 ± 1.6	0.404
CAM (mg/kg)^a^	15.7 ± 3.0	15.5 ± 2.9	0.794
Laboratory data (at the start of treatment)			
Alb (g/dL)^a^	3.7 ± 0.3	3.8 ± 0.4	0.328
Cr (mg/dL)^c^	0.7 (0.64–0.83)	0.75 (0.61–0.85)	0.960
AST (U/L)	26.5 ± 16.6	28.9 ± 9.3	0.614
ALT (U/L)^a^	17.8 ± 18.1	19.4 ± 9.2	0.728
CRP (mg/dL)^c^	0.16 (0.05–0.9)	0.2 (0.1–0.46)	0.758
WBC (/µL)^a^	6589 ± 1425	6033 ± 1212	0.178
Neutrophil (/µL)^a^	4294 ± 1418	3974 ± 1034	0.413
Evaluation of chest CT			
CT scores (at the start of treatment)^a^	14.8 ± 5.2	15.7 ± 4.8	0.578

Data are presented as ^a^ mean ± SD, ^b^ n (%) or ^c^ median (range quartile)

†: Both *Mycobacterium avium* and *Mycobacterium intracellulare* were detected in two cases

BMI, body mass index; *M.avium*,* Mycobacterium avium*; *M.intracellulare*,* Mycobacterium intracellulare*; NB, nodular bronchiectasis; FC, fibrocavitary; RFP, rifampicin; EB, ethambutol; CAM, clarithromycin; Cr, creatinine; AST, asparate aminotransferase; ALT, alanine aminotransferase; CRP, C-reactive protein; WBC, white blood cell

## Clinical course

**Fig. 1:**
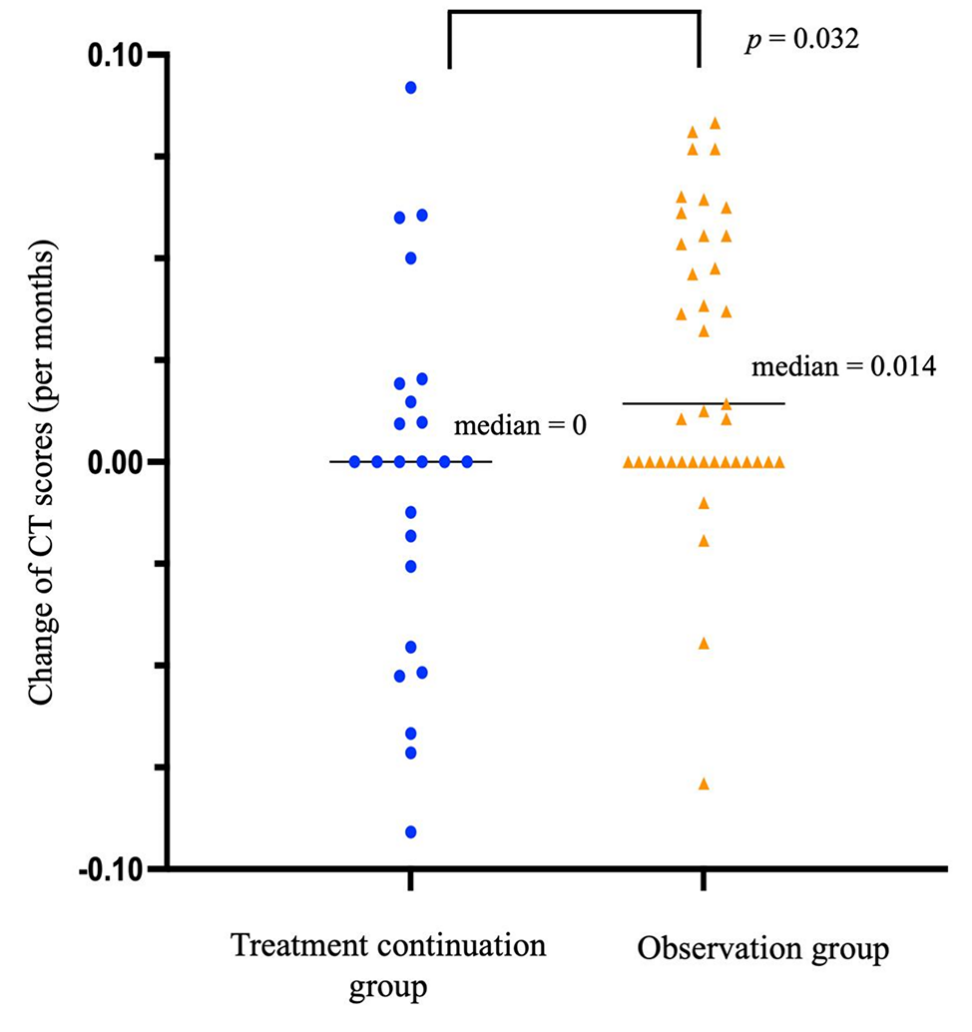
Change of CT scores between the treatment continuation group and the observation group CT, computed tomography

Treatment efficacy was compared between the time of diagnosis and the final outpatient visit in both the treatment continuation and observation groups (Table 3). The treatment continuation group showed greater improvement in symptoms change (*p* < 0.001) and maintenance of sputum conversion (*p* < 0.001) at the final outpatient visit. Follow-up CT images were not obtained for one patient in the treatment continuation group and 12 patients in the observation group. In the treatment continuation group, the change of CT scores (per month) was low (*p* = 0.032) (Fig. 1). Rates of CT scores improvement at the final outpatient visit was higher in treatment continuation group (*p* = 0.013). When comparing the two-drug and the three-drug regimens (Table 4), the two-drug regimen demonstrated non-inferiority in terms of symptoms change, sputum conversion rate, and CT scores. Two patients who achieved sputum conversion from each group were referred to another hospital after the end of treatment, hence, only 7 patients in each group were being followed up. No macrolide-resistant cases were observed after treatment in either regimen group.

**Table 3:** Clinical course in the treatment continuation group and the observation group

Parameters	Treatment continuation group (*n* = 28)	Observation group (*n* = 56)	*p*-value
Age (at diagnosis, years) ^a^	77 (76ー79)	78 (76ー84)	0.289
Age (at final visit, years) ^a^	83 (80ー86)	83 (80ー88)	0.458
Gender (%Male) ^b^	5 (17%)	24 (42%)	0.029
BMI (kg/m^2^) ^c^	19.3 ± 3.0	19.5 ± 3.2	0.868
Total follow up time (months) ^a^	54 (39ー79)	42 (28ー60)	0.051
Symptoms^b^			
Presence of symptoms during the process	20 (71%)	22 (39%)	0.010
Symptoms change ^†^			
Improvement / Stable / Worsening	12 (42%) / 13 (46%) / 3 (10%)	2 (3%) / 45 (80%) / 9 (16%)	< 0.001
Sputum culture (at final visit)b			
Sputum conversion	18 (64%)	4 (7%)	< 0.001
Culture negative once or twice	9 (32%)	17 (30%)	
Culture positive	0 (0%)	8 (14%)	
No sputum culture test	1 (3%)	27 (48%)	
Evaluation of chest CT			
Change of CT scores (per months) ^a, †, ††^	0 (-0.03ー0.02)	0.01 (0ー0.06)	0.032
Improvement of CT scores ^b^	10 (38%)	5 (10%)	0.013

Data are presented as ^a^ median (range quartile), ^b^ n(%), or ^c^ mean ± SD

†: Comparison between diagnosis and final visit

††: One patient in the treatment continuation group and 12 patients in the observation group could not be evaluated without a CT for final comparison

BMI, body mass index

**Table 4:** Clinical course in the three-drug regimen and the two-drug regimen

Parameters	three-drug regimen (*n* = 16)	two-drug regimen (*n* = 12)	*p*-value
Age (at start treatment, years)^a^	78 (76–82)	80 (78–85)	0.167
Age (at final visit, years)^b^	83 (80–86)	82 (80–86)	0.954
Gender (%Male)^c^	3 (18%)	2 (16%)	> 0.999
BMI (kg/m2)^c^	19.5 ± 3.0	15.8 ± 7.8	0.161
Duration of treatment (months)^a^	30 (22–38)	17 (14–21)	< 0.001
Symptoms change ^b, †^			
Improvement	7 (43%)	7 (58%)	0.703
Sputum culture change^b^			
Sputum conversion	9 (56%)	9 (75%)	0.434
Time of sputum conversion after treatment (days)^a^	90 (62–291)	80 (53–174)	0.572
Recurrence after sputum conversion^b, ††^	3 (42%)	1 (14%)	0.6221
Followed up period after the treatment (months) ^a, ††^	21 (9–43)	16 (9–26)	0.553
Evaluation of chest CT			
CT scores (at start treatment)^c^	14.8 ± 5.2	14.8 ± 5.4	0.991
Change of CT scores (per months)^a,†, †††^	-0.02 (-0.21ー0.02)	-0.09 (-0.1ー-0.04)	0.364
Improvement of CT scores^b^	8 (50%)	10 (83%)	0.114

Data are presented as ^a^ median (range quartile), ^b^ n(%), or ^c^ mean ± SD

†: Comparison between diagnosis and end of treatment

††: Two patients who achieved sputum conversion from each group were referred to another hospital after the end of treatment. Each of the seven patients was followed up after the treatment

†††: One patient in the three-drug regimen could not be evaluated without a CT for final comparison

BMI, body mass index.

### Adverse drug reactions

Gastrointestinal disorders were the most common ADRs leading to treatment discontinuation, followed by optic neuritis, skin disorders, hepatic dysfunction, and dizziness (Table 5). All optic neuritis cases were diagnosed by an ophthalmologist and treatment was discontinued.

**Table 5:** Adverse drug reactions leading to treatment interruption

Type of side effects	Total (*n* = 46)	three-drug regimen (*n* = 32)	two-drug regimen (*n* = 14)
Discontinuation of drugs	18 (39%)	16 (50%)	2 (14%)
Gastrointestinal disorders	7 (15%)	6 (18%)	1 (7%)
Anorexia	6 (13%)	5 (15%)	1(7%)
Nausea	1 (2%)	1 (3%)	…
Optic neuritis	4 (8%)	4 (12%)	…
Skin disorders	3 (6%)	3 (9%)	…
Hepatic dysfunction	2 (4%)	1 (3%)	1 (7%)
Dizziness	2 (4%)	2 (6%)	…
Fever	1 (2%)	1 (3%)	…
General malaise	1 (2%)	1 (3%)	…
Headache	1 (2%)	1 (3%)	…

Data are presented as n (%)

## Discussion

This study investigated the tolerability and therapeutic efficacy of treatments in real-world clinical practice for elderly MAC-PD patients aged ≥ 75 years. The two-drug regimen with EB and macrolide without RFP was associated with treatment tolerability. Both respiratory symptoms and CT scores significantly improved in the treatment continuation group compared with those in the observation group. Sputum conversion rate was also higher in the treatment continuation group. Treatment with the two-drug regimen was as effective as that with the three-drug regimen and was not associated with the development of macrolide resistance.

Studies on MAC-PD treatment in elderly patients are limited [15–17]. According to these studies, treatment success rates ranged from 55 to 70%, and the 60% success rate in this study was also comparable. Mori et al. reported that it is difficult to predict drug tolerability based on patient background and disease type before treatment [15], which aligned with our findings that patient background and laboratory findings were not associated with treatment continuation, suggesting the difficulty in predicting the tolerability of elderly MAC-PD patients before treatment. The two-drug regimen was the only clinical factor associated with tolerability. The continuation rate of the two-drug regimen was as high as 85%, indicating that the two-drug combination was a well-tolerated regimen for elderly patients. In addition, patients who were treated with three-drug regimen for more than 12 months did not discontinue treatment after 12 months. The good tolerability of two-drug regimen is thought to have a significant impact in the early stages of treatment. Also, AZM has been available for health insurance treatment since 2020 in Japan, and CAM was often used. Only two patients switched from CAM to AZM without interruption. These results suggest that AZM may be more tolerable than CAM, and are consistent with previous reports [18]. 

ADRs, especially gastrointestinal disorders, were frequent in previous study of elderly MAC-PD patients [10]. Consistently, gastrointestinal disorders were most common with the three-drug regimen in this study. Skin disorders, hepatic dysfunction, fever, general malaise which may be related to RFP were also observed. Marmor et al. reported that RFP was primarily responsible for ADRs leading to discontinuation and concluded that treatment without RFP may be more tolerable with fewer ADRs [19]. Our findings support this, showing that RFP was probably associated with ADRs leading to treatment discontinuation, and regimens without RFP improved tolerability. And then, more interruptions due to optic neuritis were observed in the three-drug combination group. Although the mechanism is not clear, it is possible that the combination of RFP with EB may have increased the frequency of optic neuritis [20]. In this study, there were no cases who were treated with triazole antifungal drug, antiviral drug, or other drug that is contraindicated in combination with RFP. However, in the elderly patients, RFP is still considered difficult to use due to interactions with drugs used to treat comorbidities.

Regarding treatment efficacy, the treatment continuation group was superior to the observation group in terms of symptoms change, sputum conversion rate, and CT scores, even after treatment completion. However, many patients in the observation group had fewer symptoms and underwent less frequent sputum testing, indicating the limitation of a simple retrospective comparison between the two groups. Regarding the efficacy of the two-drug regimen, Miwa et al. reported the non-inferiority and slightly superiority of the two-drug regimen to the three-drug regimen in terms of sputum conversion rate, symptoms change, and CT findings [11], consistent with our study. The slightly better therapeutic effect of the two-drug regimen is thought to be due to the lack of macrolide serum level reduction caused by RFP co-administration. The long treatment period of the three-drug regimen was thought to be related to the low rate of sputum conversion. Additionally, after sputum-conversion with two-drug regimen, no cases of macrolide resistance were observed after sputum conversion. The results were consistent with a previous study [21]. 

Given the potential suppressive property of rifabutin in CAM resistance in HIV-positive disseminated MAC disease, RFP has been used in combination as standard therapy [22]. However, it remains unclear whether the concomitant use of RFP contributes to the suppression of macrolide resistance in MAC-PD. Although the RFP combination is the current standard therapy, RFP does not achieve blood concentrations that maintain effective antibacterial activity against MAC, even at doses as high as 50 mg/kg/day [23]. Furthermore, for macrolide resistance suppression, Ito et al. indicated a lack of evidence for the efficacy of the RFP combination [21]. Ultimately, the results of an ongoing U.S. randomized controlled trial (Clinical Trials. gov Identifier: NCT03672630) comparing the two-drug regimen (EB and AZM) and three-drug regimen (EB, AZM, and RFP) should be carefully interpreted to determine whether the RFP combination can suppress macrolide resistance. Therefore, for elderly MAC-PD patients who are concerned about tolerability but require treatment due to persistently positive sputum cultures or progressive structural changes in the lungs due to cavity formation [24, 25], one option would be to first confirm the tolerability of EB and macrolides and then use RFP in combination.

This study had several limitations. First, the small sample size and retrospective nature of the study, conducted at only two sites, limit the generalizability of the findings. Second, follow-up CT was not performed in some cases, which may be related to the inadequate evaluation of the CT images. Third, treatment adjustments and discontinuations were determined based on the judgment of each respiratory physician and were not standardized, with treatment adjustments made only in the two-drug regimen group. A large, multicenter, prospective study is necessary to investigate the appropriate drug therapy for elderly MAC-PD patients.

## Conclusion

Our study highlights that the two-drug regimen with EB and macrolides without RFP is considered well-tolerated and effective in elderly patients with MACP-PD. Prioritizing the continuation of EB and macrolides appears to be a key factor in the treatment of elderly MAC-PD patients.

## Data Availability

The data supporting this study are available from the corresponding author upon reasonable request.

## Abbreviations

ADRs: Adverse drug reactions
ATS: American Thoracic Society
AZM: Azithromycin
BMI: Body mass index
CAM: Clarithromycin
CT: Computed tomography
EB: Ethambutol
FC: Fibrocavitary
MAC: *Mycobacterium avium* complex;
MAC-PD: *Mycobacterium avium* complex pulmonary disease
NB: Nodular bronchiectasis
NTM: Nontuberculous mycobacteria
PD: Pulmonary disease
RFP: Rifampicin
